# Machine Learning for Detecting Atrial Fibrillation from ECGs: Systematic Review and Meta-Analysis

**DOI:** 10.31083/j.rcm2501008

**Published:** 2024-01-08

**Authors:** Chenggong Xie, Zhao Wang, Chenglong Yang, Jianhe Liu, Hao Liang

**Affiliations:** ^1^Hunan Provincial Key Laboratory of TCM Diagnostics, Hunan University of Chinese Medicine, 410208 Changsha, Hunan, China; ^2^School of Acupuncture and Tui-na and Rehabilitation, Hunan University of Chinese Medicine, 410208 Changsha, Hunan, China; ^3^School of Chinese Medicine, Hunan University of Chinese Medicine, 410208 Changsha, Hunan, China; ^4^Cardiovascular Department, the First Hospital of Hunan University of Chinese Medicine, 410021 Changsha, Hunan, China

**Keywords:** machine learning, atrial fibrillation, ECG, meta-analysis

## Abstract

**Background:**

Atrial fibrillation (AF) is a common arrhythmia that can result in adverse cardiovascular outcomes but is often difficult to detect. The use of machine learning (ML) algorithms for detecting AF has become increasingly prevalent in recent years. This study aims to systematically evaluate and summarize the overall diagnostic accuracy of the ML algorithms in detecting AF in electrocardiogram (ECG) signals.

**Methods:**

The searched databases included PubMed, Web of Science, Embase, and Google Scholar. The selected studies were subjected to a meta-analysis of diagnostic accuracy to synthesize the sensitivity and specificity.

**Results:**

A total of 14 studies were included, and the forest plot of the meta-analysis showed that the pooled sensitivity and specificity were 97% (95% confidence interval [CI]: 0.94–0.99) and 97% (95% CI: 0.95–0.99), respectively. Compared to traditional machine learning (TML) algorithms (sensitivity: 91.5%), deep learning (DL) algorithms (sensitivity: 98.1%) showed superior performance. Using multiple datasets and public datasets alone or in combination demonstrated slightly better performance than using a single dataset and proprietary datasets.

**Conclusions:**

ML algorithms are effective for detecting AF from ECGs. DL algorithms, particularly those based on convolutional neural networks (CNN), demonstrate superior performance in AF detection compared to TML algorithms. The integration of ML algorithms can help wearable devices diagnose AF earlier.

## 1. Introduction

Atrial fibrillation (AF) is one of the most common arrhythmias with increasing prevalence, and it is often difficult to detect early due to its asymptomatic and paroxysmal presentation [1]. Detection of AF is mainly based on the electrocardiogram (ECG), which is challenging due to the risk of missed detection. AF is characterized by the disappearance of the P wave in each ECG lead, the replacement of the P wave by the F wave, and the atrial frequency of 350 to 600 times per minute with an irregular R-R interval. Cardiovascular adverse events such as heart failure and thromboembolism caused by AF can lead to increased morbidity and mortality [2]. Detecting paroxysmal AF is difficult with intermittent ECGs. Analyzing a large number of ECG signals generated by 24-hour Holter monitoring to detect AF requires significant human resources. How to timely detect AF and reduce the risk of missed detection while lowering medical costs remains challenging [3].

Machine learning (ML) is a subset of artificial intelligence (AI) that enables computers to learn from data and make predictions. Traditional machine learning (TML) algorithms, such as decision trees and support vector machines (SVM), require manual feature engineering. In contrast, deep learning (DL) algorithms, a new type of ML, use neural networks to automatically learn from data, making it ideal for unstructured data such as medical images and clinical text. DL has become a dominant approach within AI, revolutionizing various medical applications [4]. However, DL has drawbacks such as high computational complexity, poor portability, high hardware requirements, and difficulty in meeting the requirements of ordinary CPUs (Central-Processing-Units), making it challenging to apply DL algorithms on wearable devices.

ML has been used in recent years to analyze ECG signals for the detection of AF, with a focus on improving accuracy, and is now been better applied and developed in medical diagnosis and treatment [5]. With the continuous updating of technology and the continuous expansion of public datasets, various ML algorithms have been gradually applied for the clinical diagnosis of arrhythmias. For example, TML algorithms represented by SVM and DL algorithms represented by convolution neural network (CNN) and long short-term memory (LSTM) have been widely used to analyze ECG signals to detect AF [6]. Some new algorithms that combine DL and other methods have achieved better performance, such as Deepaware algorithms and DeepBeat [7, 8]. In addition, wearable devices such as smartwatches equipped with ECG sensors offer a convenient and non-invasive means of collecting ECG signals for AF detection. These devices provide a wealth of data that can be used to train and improve ML algorithms for better performance. Overall, ML has great potential to improve the efficiency and accuracy of AF detection.

However, the results of several studies have been inconsistent because various factors may affect the performance of the algorithms [7, 9, 10, 11, 12, 13], such as the selection of signals, the use of TML or DL algorithms, and the included datasets. It is, therefore, necessary to systematically review and meta-analyze ML in the detection of AF in ECGs, summarize sensitivity, specificity, and area under curve (AUC) of various algorithms, and investigate whether the results are related to the above factors so as to evaluate the application of ML in the detection of AF in ECGs.

## 2. Methods

This study was performed in accordance with the guidelines of Preferred Reporting Items for Systematic Reviews and Meta-Analyses (PRISMA-DTA) [14] and was part of work registered in INPLASY (No. 202310047). All analyses were based on previously published studies; thus, no ethical approval and patient consent was required.

### 2.1 Search Strategy

A comprehensive search strategy was designed and executed within PubMed, Web of Science, Embase, and Google Scholar databases from their inception until January 30, 2023. A combination of subject words and keywords was used to formulate this search strategy, with adjustments made to account for differences in the various databases. As an example, the fewer keywords or corresponding Medical Subject Headings (MeSH) were used for searching ((Atrial Fibrillation [MeSH Terms]) or (Auricular Fibrillation [Title/Abstract])) and ((Machine Learning [MeSH Terms]) or (Algorithms [Title/Abstract]) or (Artificial Intelligence [Title/Abstract]) or (Deep Learning [Title/Abstract])) and ((Electrocardiography [MeSH Terms]) or (Electrocardiography (EKG) [Title/Abstract]) or (ECG [Title/Abstract])). The complete search strategy is shown in **Supplementary Fig. 1**.

**Fig. 1.**
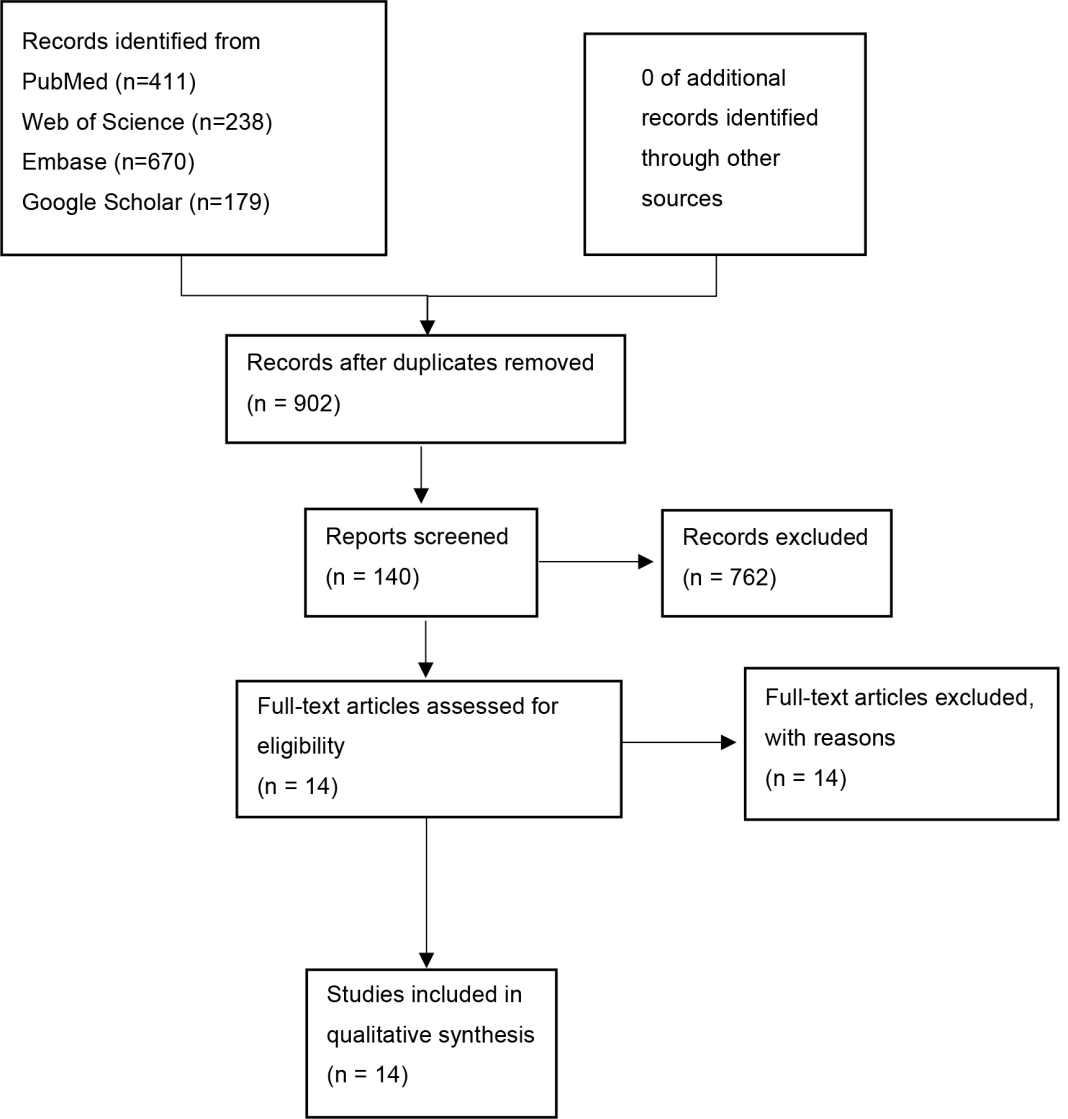
**Flow chart of the selection process**.

One investigator (ZW) designed and conducted the search strategy using input from the study’s principal investigator (CGX). The literature search process is shown in Fig. 1. From the initial pool of 902 studies, 140 studies were screened based on the assessment of their titles and abstracts. A full-text review was then conducted, resulting in the exclusion of 762 studies. Ultimately, 14 studies were included in the quality assessment and meta-analysis [15, 16, 17, 18, 19, 20, 21, 22, 23, 24, 25, 26, 27, 28].

### 2.2 Study Selection

The two primary reviewers (ZW and CGX) screened abstracts and titles of the studies to assess their eligibility for inclusion in the meta-analysis. The criteria for eligibility were: (1) studies that focused on the detection of AF, (2) studies that developed AI models using ML algorithms, and (3) studies that utilized ECG signals obtained from AF patients or available AF datasets. The exclusion criteria were: (1) studies that only evaluated cardiovascular parameters without considering the disease status, (2) studies with a small sample size of patients (≤10) or ECG signals (≤100), (3) studies that did not report the quantitative performance metrics such as sensitivity and specificity, (4) studies of AF complicated with other diseases, (5) studies that used incomplete ECG signals and only analyzed specific ECG characteristics, and (6) the dataset used for the algorithm in studies must come from multiple different databases or patients or be reasonably split between training and validation datasets. Finally, 14 studies were deemed eligible for inclusion in the quality assessment and meta-analysis.

### 2.3 Data Extraction and Quality Assessment

Data parameters were determined before the literature search was extracted in the selected studies. We extracted the following information, if possible, from each study: authors, year of publication, study name, study tasks, datasets and their availability (proprietary datasets or public AF datasets), ML algorithm types, and performance measures (AUC, sensitivity, specificity). We evaluated the risk of bias in individual studies using the quality assessment of diagnostic accuracy studies-2 (QUADAS-2) tool [29].

### 2.4 Data Synthesis

We aimed to summarize the performance of ML algorithms that could be used to detect AF and present the results in the form of summary statistics. We extracted the number of true positive (TP), false positive (FP), false negative (FN), and true negative (TN) data from these studies. If these parameters were unavailable, they were calculated based on sample size and performance indicators such as sensitivity and specificity. In the event that these parameters could not be calculated, the study was excluded.

To mitigate the heterogeneity among the studies in the meta-analysis, meta-regression, and subgroup analysis were performed according to the type of algorithm (TML or DL), datasets and their availability (proprietary datasets or public datasets), and the number of used datasets (using a single dataset and multiple datasets).

### 2.5 Statistical Analysis

Bivariate and hierarchical summary receiver operating characteristic (HSROC) models were performed to jointly estimate sensitivity, specificity, and AUC. Meta-regression analysis and subgroup analysis were performed according to the above subgroups. All statistical analyses were performed using Revman version 5.4 (Cochrane Collaboration, London, UK), R version 4.2.1 (mada, lme4, lmtest, and msm packages, Vienna, Austria), and Stata version 14.1 (metandi and midas packages, College Station, TX, USA).

## 3. Results

### 3.1 Characteristics of Included Studies

Fourteen studies were included in the quality assessment and meta-analysis [15, 16, 17, 18, 19, 20, 21, 22, 23, 24, 25, 26, 27, 28]. The characteristics of the studies are shown in **Supplementary Table 1**.

Ten studies [16, 18, 19, 21, 23, 24, 25, 26, 27, 28] used DL, while only 4 studies [15, 17, 20, 22] used TML. Among the included studies, the CNN algorithm was the most commonly used. In addition, a few studies [23, 28] used a combination of multiple algorithms.

Ten studies [15, 16, 17, 19, 20, 22, 23, 24, 25, 27] used public datasets alone or in combination with proprietary datasets, and many of them used the MIT-BIH AF database. The MIT-BIH AF database was provided by the Massachusetts Institute of Technology, which contained long-term ECG records of 25 patients with paroxysmal AF for use by researchers. The remaining 4 studies [18, 21, 26, 28] only used proprietary datasets, which usually came from ECG signals collected by medical institutions or smart wearable devices at home.

Eight studies [15, 17, 20, 22, 24, 25, 26, 27] used a single dataset as a data source, and only 6 studies [16, 18, 19, 21, 23, 28] used multiple datasets as a data source. While the use of a single dataset reduced the differences among data, it could also limit the generalizability of ML performance indicators to other datasets.

### 3.2 Quality Assessment

Regarding the assessment of bias risk using QUADAS-2, 14 relevant studies were evaluated across 4 domains: patient selection, index test, reference standard, and flow and timing [15, 16, 17, 18, 19, 20, 21, 22, 23, 24, 25, 26, 27, 28].

For each domain assessed by the QUADAS-2 tool, the number of studies classified as having a high, unclear, or low risk of bias are as follows: patient selection (4 studies with high risk of bias, 4 with unclear risk, and 6 with low risk), index test (0 with high risk, 6 with unclear risk, and 8 with low risk), reference standard (0 with high risk, 7 with unclear risk, and 7 with low risk), and flow and timing (1 with high risk, 2 with unclear risk, and 11 with low risk). The detailed quality assessment of the included studies using the QUADAS-2 tool is shown in **Supplementary Fig. 2**.

### 3.3 ML Algorithms and Detection of Atrial Fibrillation in ECG

**Fig. 2.**
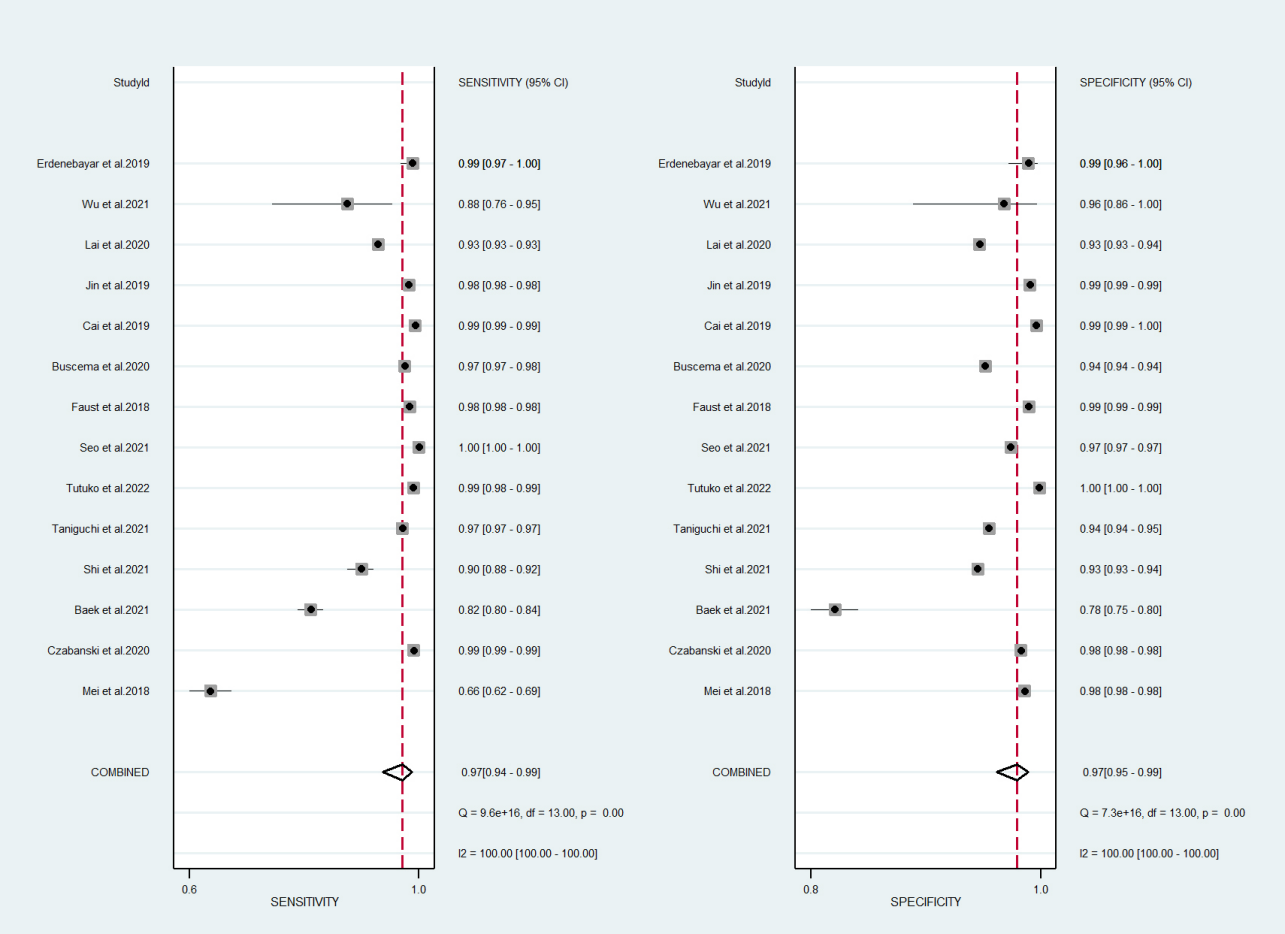
**Forest plot of machine learning for atrial fibrillation detection in electrocardiogram**. CI, confidence interval.

In total, 14 studies were included in the quantitative meta-analysis, and the algorithms had a high performance in the detection of AF in ECGs [15, 16, 17, 18, 19, 20, 21, 22, 23, 24, 25, 26, 27, 28]. The forest plot of meta-analysis revealed high diagnostic performance for AF detection, with a sensitivity of 97% (95% confidence interval [CI]: 0.94–0.99) and a specificity of 97% (95% CI: 0.95–0.99) (Fig. 2).

**Fig. 3.**
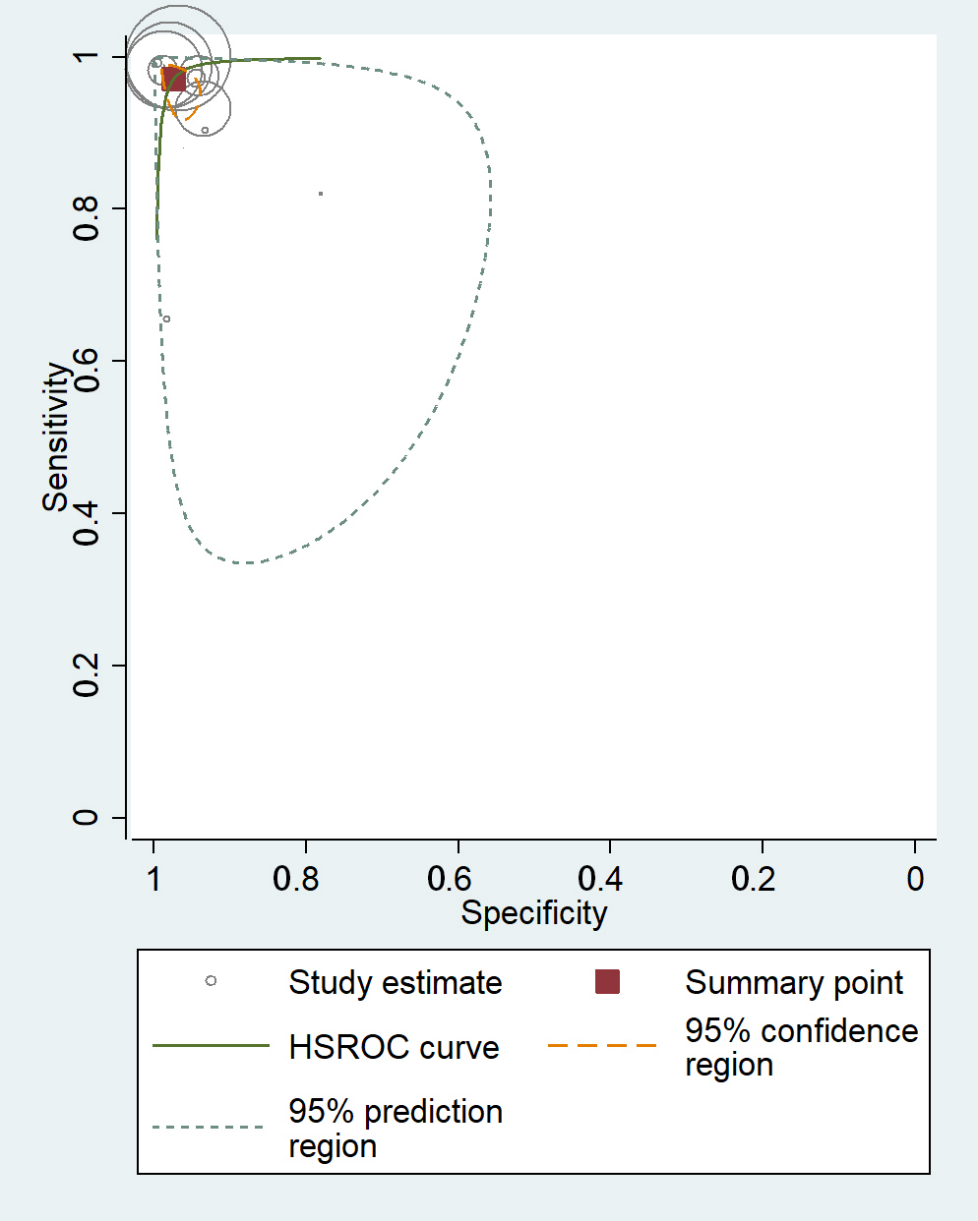
**Receiver operating characteristics curves of machine learning for atrial fibrillation detection in electrocardiogram**. HSROC, hierarchical summary receiver operating characteristic.

The receiver operating characteristics (ROC) curves of meta-analysis showed that the majority of the studies had a high level of sensitivity and specificity, demonstrating their potential to effectively detect AF in ECG signals (Fig. 3).

High heterogeneity was observed among the included studies, evident from the Q-value of 9.6 × ${\mathrm{10}}^{16}$ for sensitivity and 7.3 × ${\mathrm{10}}^{16}$ for specificity. A meta-regression analysis and subgroup analyses were conducted to explore potential sources of heterogeneity.

### 3.4 Meta-Regression

**Fig. 4.**
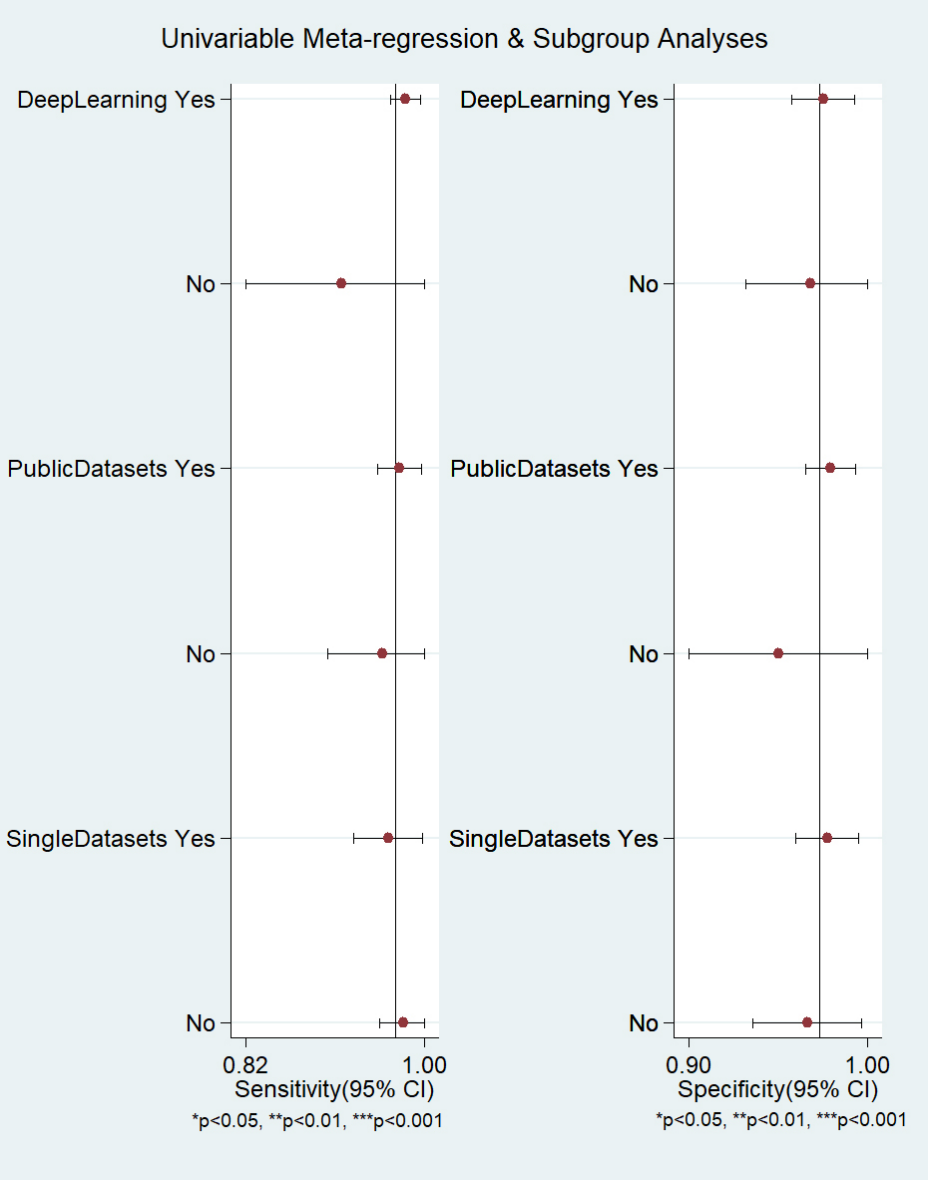
**Meta-regression and subgroup analysis of machine learning for atrial fibrillation detection in electrocardiogram**. CI, confidence interval.

According to the characteristics of the studies, we selected 3 factors (ML algorithm types, datasets, their availability, and number of used datasets) to conduct meta-regression and subgroup analysis for the included studies. The results showed that the heterogeneity of ML algorithm types (deep learning in Fig. 4) sensitivity and specificity was not significant (*p* > 0.05). In addition, the heterogeneity of datasets and their availability (Public Datasets in Fig. 4) and number of used datasets (Single Datasets in Fig. 4) specificity was not significant (*p* > 0.05). Therefore, ML algorithm types, datasets, and their availability and number of used datasets could not be the reasons for the high heterogeneity of the meta-analysis.

### 3.5 Comparison of ML Algorithm Types

In the quantitative meta-analysis of included studies, DL algorithms achieved an AUC of 100% (95% CI: 0.99–1.00), while TML algorithms attained an AUC of 0.98 (95% CI: 0.97–0.99). DL algorithms exhibited higher performance in the detection of AF in ECGs as compared to TML algorithms. The results of the meta-analysis showed that DL algorithms had a higher diagnostic sensitivity as compared to TML algorithms.

**Fig. 5.**
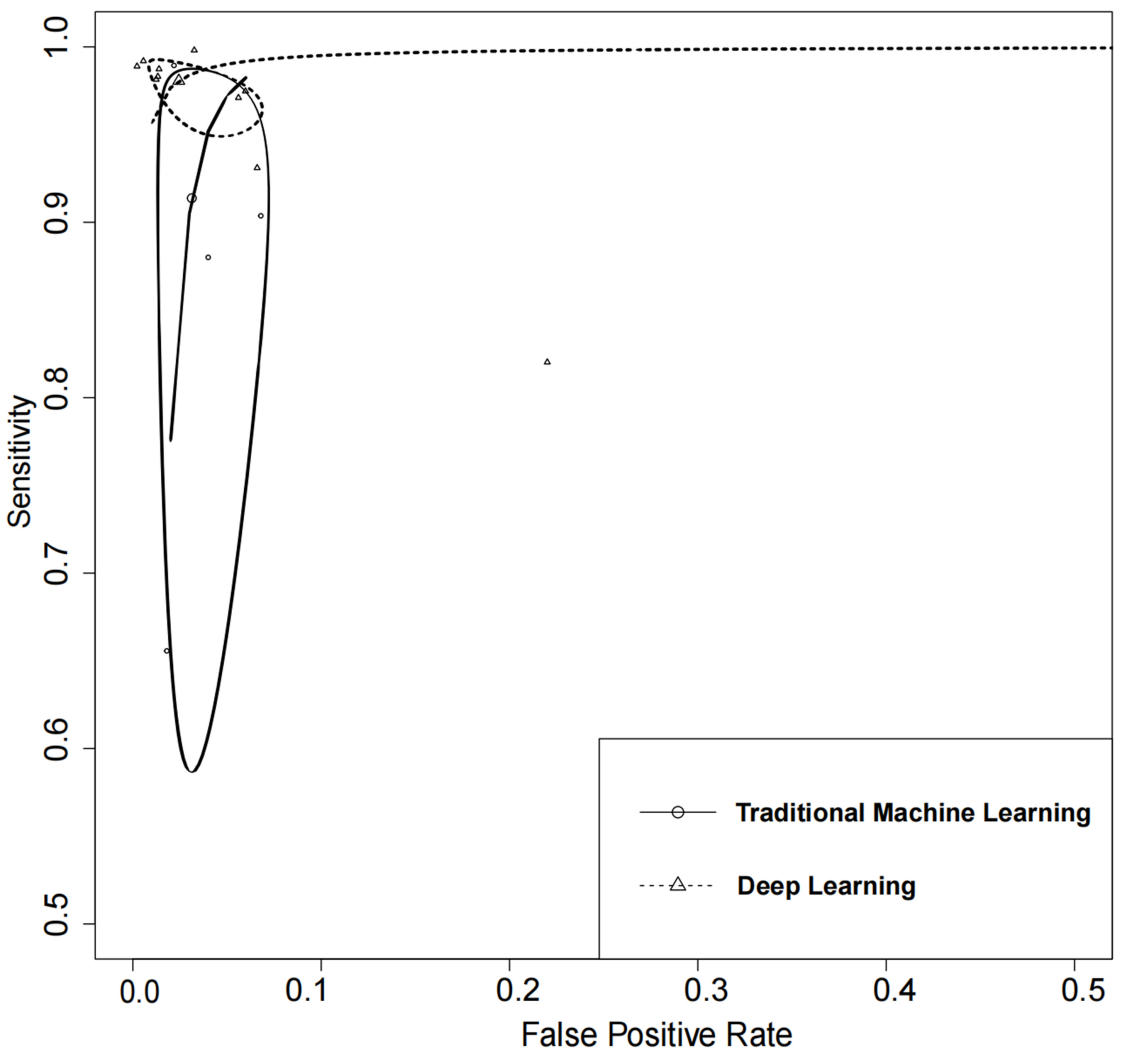
**Summary of receiver operating characteristics curves of traditional machine learning and deep learning**.

Although the difference in specificity between them was not significant (*p* > 0.05), DL algorithms demonstrated a better overall performance (Fig. 5).

### 3.6 Comparison of the Datasets Types and Their Availability

The performance of using public datasets yielded an AUC of 100% (95% CI: 0.99–1.00), sensitivity of 97% (95% CI: 0.94–0.99), and specificity of 98% (95% CI: 0.96–0.99). In contrast, using proprietary datasets achieved an AUC of 0.99 (95% CI: 0.97–0.99), sensitivity of 96% (95% CI: 0.87–0.99), and specificity of 95% (95% CI: 0.83–0.99). When considering the number of datasets used, using single datasets had an AUC of 97% (95% CI: 0.96–0.98), sensitivity of 92% (95% CI: 0.83–0.97), and specificity of 93% (95% CI: 0.81–0.98). In contrast, using multiple datasets achieved an AUC of 100% (95% CI: 0.98–1.00), sensitivity of 98% (95% CI: 0.93–0.99), and specificity of 97% (95% CI: 0.90–0.99).

**Fig. 6.**
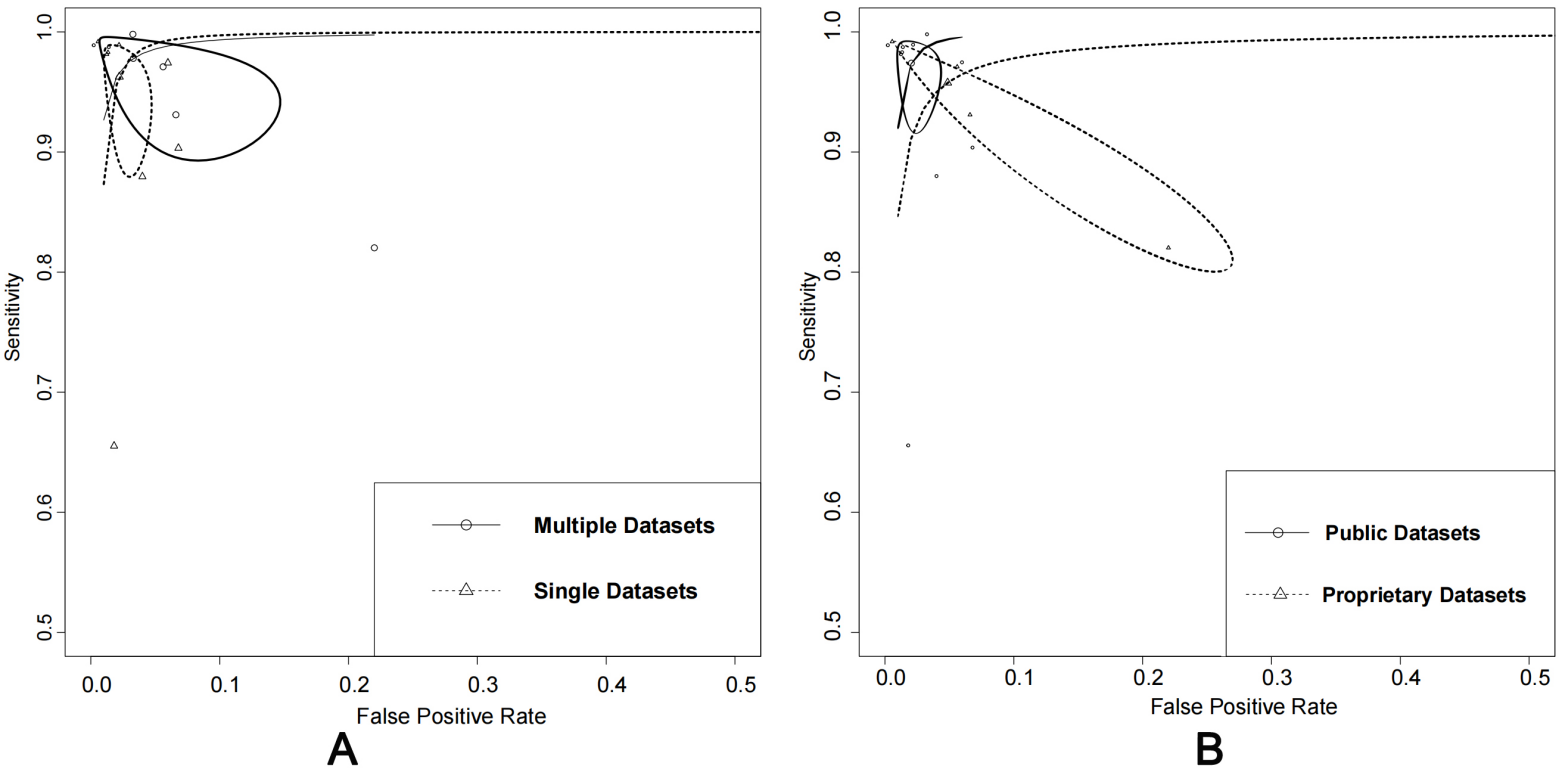
**Comparison: single vs. multiple datasets and public vs. proprietary datasets**. (A) Summary of receiver operating characteristics curves of single datasets vs. multiple datasets. (B) Summary of receiver operating characteristics curves of using public datasets alone or in combination with proprietary datasets.

The analysis of the impact of dataset types and their availability on algorithm performance showed that the use of multiple datasets and public datasets demonstrated slightly better performance. However, there was no significant difference (*p* > 0.05) between using single datasets or multiple datasets and between using public datasets alone or in combination with proprietary datasets (Fig. 6A,B). These findings suggest that the availability and number of datasets are important factors in testing ML algorithms for AF detection and that the performance benefits of using multiple or public datasets may be relatively better.

### 3.7 Publication Bias

The *p-value*, obtained by the Deeks’ Funnel Plot Asymmetry Test method, was 0.28, so there was no publication bias.

## 4. Discussion

In this systematic review and meta-analysis, the performance of ML designed for AF detection in ECGs was comprehensively evaluated. This research aimed to provide insights into the development of these algorithms through the synergistic synthesizing of sensitivity and specificity of each study and the conduct of meta-regression and subgroup analysis.

Three main sources of data for ML algorithms were identified: (1) public ECG databases, such as the MIT AF database [24, 27, 30], (2) ECG signals collected in medical institutions [21, 26], and (3) ECG signals collected by wearable or implantable devices [28]. The first two methods have lower interference with the ECG signal acquisition environment and are easier to obtain high-quality ECG signals, resulting in generally excellent accuracy of the algorithms. The third method collects ECG or photoelectric signals often influenced by other factors such as electromyography (EMG) signals. However, while implementing a particular algorithm is largely convenient for non-medical workers, the accuracy of the relevant algorithms involved needs to be improved [31].

In terms of dataset usage, ML algorithms can be trained using single or multiple datasets. The use of single datasets reduces performance differences caused by various data sources but lacks external validation, leading to a lack of credibility in performance. In many studies, the use of the same ML algorithm in different datasets has been showed to produce performance variations [16, 18, 32]. Data preprocessing is one of the methods to obtain high-quality ECG signals, which can transform the data into a form that is more accessible to ML, such as filtering, noise removal, and feature extraction [3]. Specific operations in each study can greatly affect the final performance indicators of ML algorithms.

ECG signals typically consist of the P wave, QRS wave group, and T wave, among others. While the extraction of specific waves or wave groups, such as the P wave, has been explored as a way to detect AF through ML, the performance of this approach has not been found to be optimal [33, 34]. Further optimization of algorithms and improvement of data extraction methods are required to enhance the accuracy.

Similarly, the performance of different ML algorithms differs. DL algorithms are generally superior to TML algorithms and are widely used in the detection of AF. DL algorithms represented by CNN are the most applicable. In some studies, CNN algorithms have been combined with other DL algorithms and even combined with TML algorithms in order to produce a new model [23, 35]. The combination of multiple ML algorithms can potentially become a novel and effective ML method for AF detection. The interpretability of DL is a major challenge to application in clinical practice. Despite the high accuracy of DL algorithms, medical workers could not explain the results to patients without an adequate explanation [36]. In clinical practice, the interpretability of DL algorithms remains a challenge as they are considered black boxes, and the results generated by them cannot be fully explained [37].

ML algorithms are primarily aimed at distinguishing AF ECG signals from non-AF signals and further classifying AF signals into different subtypes, such as paroxysmal atrial fibrillation (PAF) and persistent atrial fibrillation (PeAF). Furthermore, as clinical AF patients may also have other types of arrhythmia diseases or other types of cardiovascular diseases, the use of ML to distinguish AF from other types of arrhythmias, and even from other types of cardiovascular diseases, is a challenging task [38].

The other important goal of ML algorithms is to actively predict the onset time of AF. For healthy individuals, AF has not yet occurred, and they need to rely on ML to predict the risk of AF. In the case of a risk, it is necessary to continue to predict the time of future AF episodes. For individuals who have experienced AF, they need to rely on ML to predict the time of the next AF episode [13]. Due to the high similarity between pre-attack ECG signals and normal ECG signals, it is still difficult to actively predict the time of onset of AF, which requires research.

In order to address the limitations of large equipment in medical institutions, the use of portable and wearable or implantable ECG equipment has become a popular research area in medical and AI fields. Portable and wearable ECG devices play a significant role in the detection of AF, as they can effectively reduce the burden on the medical system by providing early screening for AF and its potential patients and providing internet data support for sharing relevant data between medical professionals and patients [39]. Our research results indicate that AI-based algorithms have the potential to revolutionize the field of AF detection and monitoring, especially when combined with portable and wearable ECG devices. The key advantage of these devices is their ability to continuously monitor patients in real-time and provide timely alerts for potential AF episodes [40]. With DL algorithms such as CNN, AF detection in these devices has reached a high level of accuracy and reliability, making them an ideal choice for large-scale applications. With the widespread use of intelligent wearable devices such as smartwatches in daily life, there is a great opportunity for improving the detection of AF with relevant algorithms [41].

The use of ML for AF detection on wearable devices presents several challenges. First, the limited processing power and memory capacity of wearable devices may hinder the deployment of complex ML algorithms, especially DL algorithms, which typically require significant resources for training and testing. Embedding ML algorithms into electronic health records (EHR) or developing ML algorithms with lower computational requirements that can be used directly for wearable devices may be an effective way to solve this problem [42]. In addition, the presence of motion artifacts and noise in ECG signals or photoplethysmogram (PPG) signals acquired by wearable devices may negatively impact the performance of ML algorithms, requiring robust signal preprocessing and noise reduction techniques [9]. Furthermore, ensuring the interpretability of wearable devices helps users and researchers to better accept relevant algorithms. Overcoming these challenges will be essential to apply ML in AF detection on wearable devices and enable widespread adoption for improved healthcare outcomes.

Real-time results can be obtained by intelligent wearable devices processing signals collected from individuals in their daily lives, but the performance may be reduced by data acquisition, preprocessing, and noise factors. The solution to this problem is the development of accurate data acquisition equipment, the invention of a lightweight data preprocessing mode, and the reduction of noise interference [31]. Currently, with continuous improvements in medical devices, the use of wearable ECG devices to monitor health can help identify potential AF patients [40]. However, existing studies demonstrated the need to improve the accuracy of AI models for wearable devices, and the development of ML algorithms that can be applied to wearable devices has become an area of active research issue.

Mobilenet is a lightweight DL model for arrhythmia classification in embedded wearable devices. This model uses multi-sensor units for data processing and classification [43]. Compared to the resnet model, mobilenet demonstrates higher efficiency, with a remarkable reduction in size from 743 MB to 76 KB (1/10,000) using model compression (TensorFlow Lite) while maintaining similar levels of accuracy [44]. Its model compression capability significantly reduces weight, making it an ideal choice for real-time AF detection in wearable devices.

A limitation of this systematic review and meta-analysis is the high heterogeneity of the included studies, which was partially addressed through meta-regression and subgroup analysis. However, inappropriate statistical analysis may have been generated during the quantitative comprehensive research.

## 5. Conclusions

In conclusion, ML algorithms perform effectively for AF detection. In terms of the type of ML algorithms, number of datasets, and dataset availability, using DL algorithms, multiple datasets, and public datasets enable better performance. DL algorithms, such as CNN, can be applied in clinical practice for AF detection in view of their superior performance. The integration of ML algorithms with wearable devices has the potential to transform the method of AF detection, enabling more accurate and personalized diagnosis, risk stratification, and therapy selection.

## Data Availability

All data generated or analyzed during this study are included in this published article.

## Supplementary Material

Supplementary material associated with this article can be found, in the online version, at https://doi.org/10.31083/j.rcm2501008.
