# Supplementary material for: Machine Learning for Detecting Atrial Fibrillation from ECGs: Systematic Review and Meta-Analysis
Source: Rev Cardiovasc Med. 2024 Jan 8;25(1):8. doi: 10.31083/j.rcm2501008 (PMC11262392; doi:10.31083/j.rcm2501008)
Supplement: Supplementary file 1 [file 2153-8174-25-1-008-s1.zip › 2153-8174-25-1-008-s1/PRISMA_2020_flow_diagram_new_SRs_v1.pdf]

PRISMA 2020 flow diagram for new systematic reviews which included searches of databases and registers only

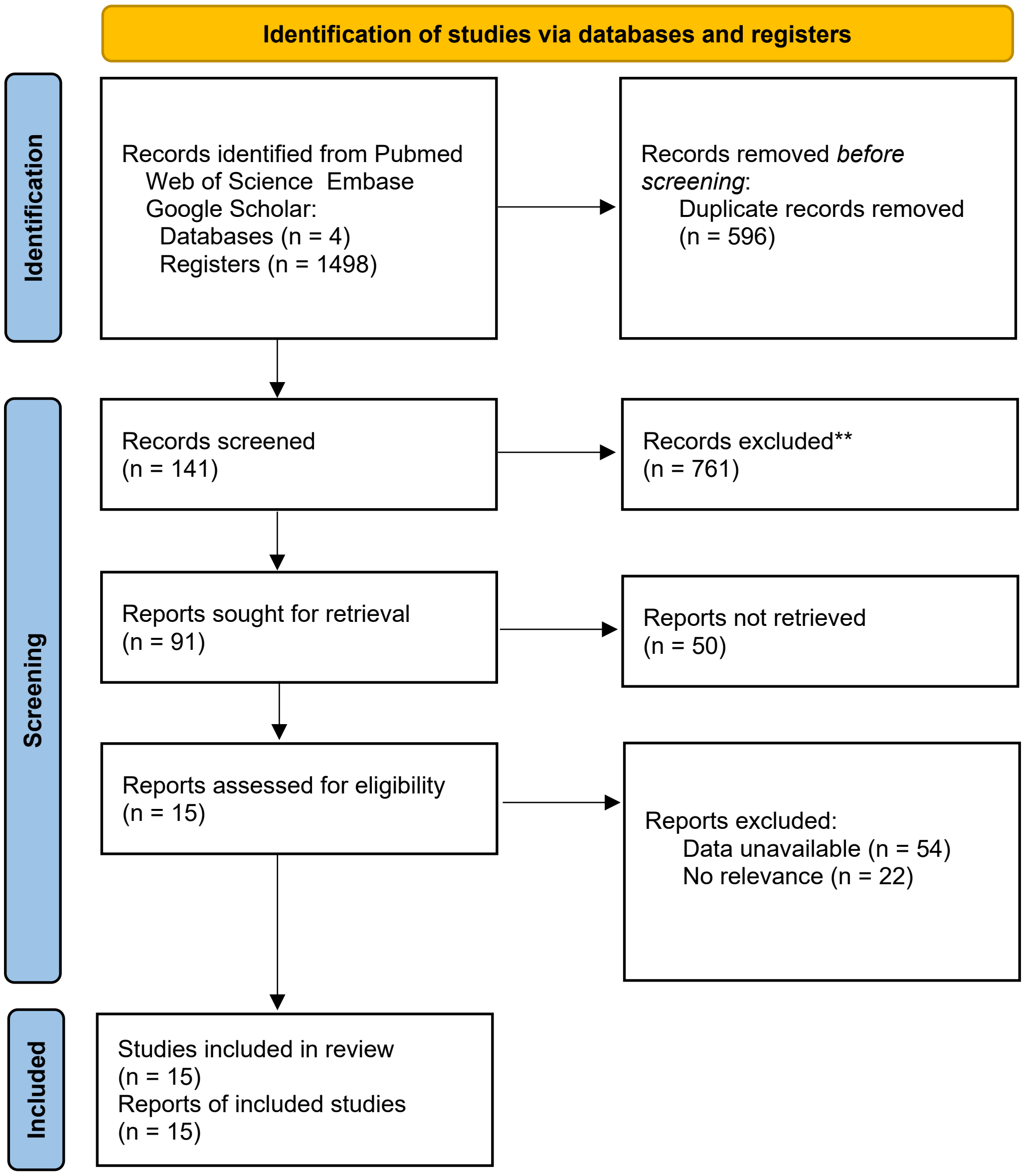

*Consider, if feasible to do so, reporting the number of records identified from each database or register searched (rather than the total number across all databases/registers).

**If automation tools were used, indicate how many records were excluded by a human and how many were excluded by automation tools.

From: Page MJ, McKenzie JE, Bossuyt PM, Boutron I, Hoffmann TC, Mulrow CD, et al. The PRISMA 2020 statement: an updated guideline for reporting systematic reviews. BMJ 2021;372:n71. doi: 10.1136/bmj.n71

For more information, visit: http://www.prisma-statement.org/
