# Supplementary material for: Machine Learning for Detecting Atrial Fibrillation from ECGs: Systematic Review and Meta-Analysis
Source: Rev Cardiovasc Med. 2024 Jan 8;25(1):8. doi: 10.31083/j.rcm2501008 (PMC11262392; doi:10.31083/j.rcm2501008)
Supplement: Supplementary file 1 [file 2153-8174-25-1-008-s1.zip › 2153-8174-25-1-008-s1/PRISMA_DTA_Checklist.pdf]

PRISMA-DTA Checklist

Section/topic	#	PRISMA-DTA Checklist Item	Reported on page #
**TITLE / ABSTRACT**			
Title	1	Machine Learning for Detecting Atrial Fibrillation from ECG: Systematic Review and Meta-analysis	Page 1
Abstract	2	Background: Atrial fibrillation (AF) is a common arrhythmia that can cause serious health consequences if left untreated. The use of machine learning (ML) algorithms for the detection of AF has become increasingly prevalent in recent years. This study aims to systematically evaluate and summarize the overall diagnostic accuracy of the ML algorithms in detecting AF in electrocardiogram (ECG) signals.<br>Methods: The searched databases included PubMed, Web of Science, Embase, and Google Scholar. The selected studies were subjected to a meta-analysis of diagnostic accuracy to synthesize the sensitivity, specificity, and AUC.<br>Results: A total of 15 studies were included in the qualitative review, and the results showed that the ML algorithms had high performance in detection of AF in ECG. The forest plot of meta-analysis showed that diagnostic sensitivity was 97% (95% CI: 0.93 - 0.98), diagnostic specificity was 97% (95% CI: 0.94 - 0.98). Compared to traditional machine learning (TML) algorithms (AUC: 98%), deep learning (DL) algorithms (AUC: 100%) showed superior performance. Using multiple datasets (AUC: 100%) resulted in better algorithm performance compared to using a single dataset (AUC: 99%). Furthermore, using public datasets alone or in combination (AUC: 99%) showed slightly better performance than using proprietary datasets (AUC: 99%).<br>Conclusions: This research find that ML algorithms are effective in detection of AF in ECG, with DL algorithms, particularly those based on convolutional neural networks (CNN), displaying the best performance. The results suggest that ML algorithms have potential to be useful tools in detection of AF.	Page 2
**INTRODUCTION**			
Rationale	3	It is necessary to systematically review and meta-analyze ML in detection of AF in ECG, and summarize sensitivity, specificity and AUC of various algorithms, and investigate whether the results are related to the above factors, so as to evaluate application of ML in detection of AF in ECG.	Page 5
Clinical role of index test	D1	State the scientific and clinical background, including the intended use and clinical role of the index test, and if applicable, the rationale for minimally acceptable test accuracy (or minimum difference in accuracy for comparative design).	Page 5
Objectives	4	To evaluate application of ML in detection of AF in ECG.	Page 5
**METHODS**			
Protocol and registration	5	INPLASY202310047 10.37766/inplasy2023.1.0047	Page 5
Eligibility criteria	6	Specify study characteristics (participants, setting, index test(s), reference standard(s), target condition(s), and study design) and report characteristics (e.g., years considered, language, publication status) used as criteria for eligibility, giving rationale.	Page 27
Information sources	7	PubMed, Web of Science, Embase, and Google Scholar databases from their inception until January 30, 2023.	Page 6
Search	8	Pubmed:((Atrial Fibrillation[MeSH Terms]) OR (Auricular Fibrillation[Title/Abstract])) AND ((Machine Learning[MeSH Terms]) OR (Algorithms[Title/Abstract]) OR (Artificial Intelligence[Title/Abstract]) OR (Deep Learning[Title/Abstract])) AND ((Electrocardiography[MeSH Terms]) OR (EKG[Title/Abstract]) OR (ECG[Title/Abstract]))<br>Web of Science:((machine learning) OR (artificial intelligence)) AND (ECG) AND (atrial fibrillation)	Page 6

PRISMA-DTA Checklist

		Google Scholar:allintitle: ("machine learning" OR"artificial intelligence"OR"deep learning") AND "atrial fibrillation"<br>Embase:('machine learning'/exp OR 'machine learning':ab,ti OR algorithms:ab,ti OR 'artificial intelligence':ab,ti OR 'deep learning':ab,ti) AND ('atrial fibrillation'/exp OR 'atrial fibrillation':ab,ti OR 'auricular fibrillation':ab,ti) AND ('electrocardiography'/exp OR electrocardiography:ab,ti OR ekg:ab,ti OR ecg:ab,ti) AND (detection:ab,ti OR diagnosis:ab,ti)	
Study selection	9	State the process for selecting studies (i.e., screening, eligibility, included in systematic review, and, if applicable, included in the meta-analysis).	Page 20
Data collection process	10	Describe method of data extraction from reports (e.g., piloted forms, independently, in duplicate) and any processes for obtaining and confirming data from investigators.	Page 7
Definitions for data extraction	11	Provide definitions used in data extraction and classifications of target condition(s), index test(s), reference standard(s) and other characteristics (e.g. study design, clinical setting).	Page 7
Risk of bias and applicability	12	We evaluated the risk of bias of individual studies using the quality assessment of diagnostic accuracy studies-2 (QUADAS-2) tool.	Page 11
Diagnostic accuracy measures	13	sensitivity, specificity ECG signals	Page 8
Synthesis of results	14	Describe methods of handling data, combining results of studies and describing variability between studies. This could include, but is not limited to: a) handling of multiple definitions of target condition. b) handling of multiple thresholds of test positivity, c) handling multiple index test readers, d) handling of indeterminate test results, e) grouping and comparing tests, f) handling of different reference standards	Page 9

Section/topic	#	PRISMA-DTA Checklist Item	Reported on page #
Meta-analysis	D2	Bivariate models and Hierarchical Summary Receiver Operating Characteristic (HSROC) models.	Page 8
Additional analyses	16	subgroup analyses and meta-regression	Page 11
**RESULTS**			
Study selection	17	Provide numbers of studies screened, assessed for eligibility, included in the review (and included in meta-analysis, if applicable) with reasons for exclusions at each stage, ideally with a flow diagram.	Page 20
Study characteristics	18	For each included study provide citations and present key characteristics including: a) participant characteristics (presentation, prior testing), b) clinical setting, c) study design, d) target condition definition, e) index test, f) reference standard, g) sample size, h) funding sources	Page 24
Risk of bias and applicability	19	Present evaluation of risk of bias and concerns regarding applicability for each study.	Page 21
Results of individual studies	20	For each analysis in each study (e.g. unique combination of index test, reference standard, and positivity threshold) report 2x2 data (TP, FP, FN, TN) with estimates of diagnostic accuracy and confidence intervals, ideally with a forest or receiver operator characteristic (ROC) plot.	Page 21
Synthesis of results	21	Describe test accuracy, including variability; if meta-analysis was done, include results and confidence intervals.	Page 21

Additional analysis	23	Give results of additional analyses, if done (e.g., sensitivity or subgroup analyses, meta-regression; analysis of index test: failure rates, proportion of inconclusive results, adverse events).	Page 22-23
**DISCUSSION**			
Summary of evidence	24	Summarize the main findings including the strength of evidence.	Page 15-16
Limitations	25	Discuss limitations from included studies (e.g. risk of bias and concerns regarding applicability) and from the review process (e.g. incomplete retrieval of identified research).	Page 15
Conclusions	26	Provide a general interpretation of the results in the context of other evidence. Discuss implications for future research and clinical practice (e.g. the intended use and clinical role of the index test).	Page 15-16
**FUNDING**			
Funding	27	This research study was supported by the grants of National Natural Science Foundation of China (No. 82274411), Science and Technology Innovation Program of Hunan Province (No. 2022RC1021), Natural Science Foundation of Hunan Province (No. 2022JJ40300).	Page 16

Adapted From: McInnes MDF, Moher D, Thombs BD, McGrath TA, Bossuyt PM, The PRISMA-DTA Group (2018). Preferred Reporting Items for a Systematic Review and Meta-analysis of Diagnostic Test Accuracy Studies: The PRISMA-DTA Statement. JAMA. 2018 Jan 23;319(4):388-396. doi: 10.1001/jama.2017.19163.

For more information, visit: **www.prisma-statement.org**.
