# Supplementary material for: Machine Learning for Detecting Atrial Fibrillation from ECGs: Systematic Review and Meta-Analysis
Source: Rev Cardiovasc Med. 2024 Jan 8;25(1):8. doi: 10.31083/j.rcm2501008 (PMC11262392; doi:10.31083/j.rcm2501008)
Supplement: Supplementary file 1 [file 2153-8174-25-1-008-s1.zip › 2153-8174-25-1-008-s1/supplementary material-V2 for reviewing.docx]

Supplementary files

Supplementary Table 1. Summary of the included studies.


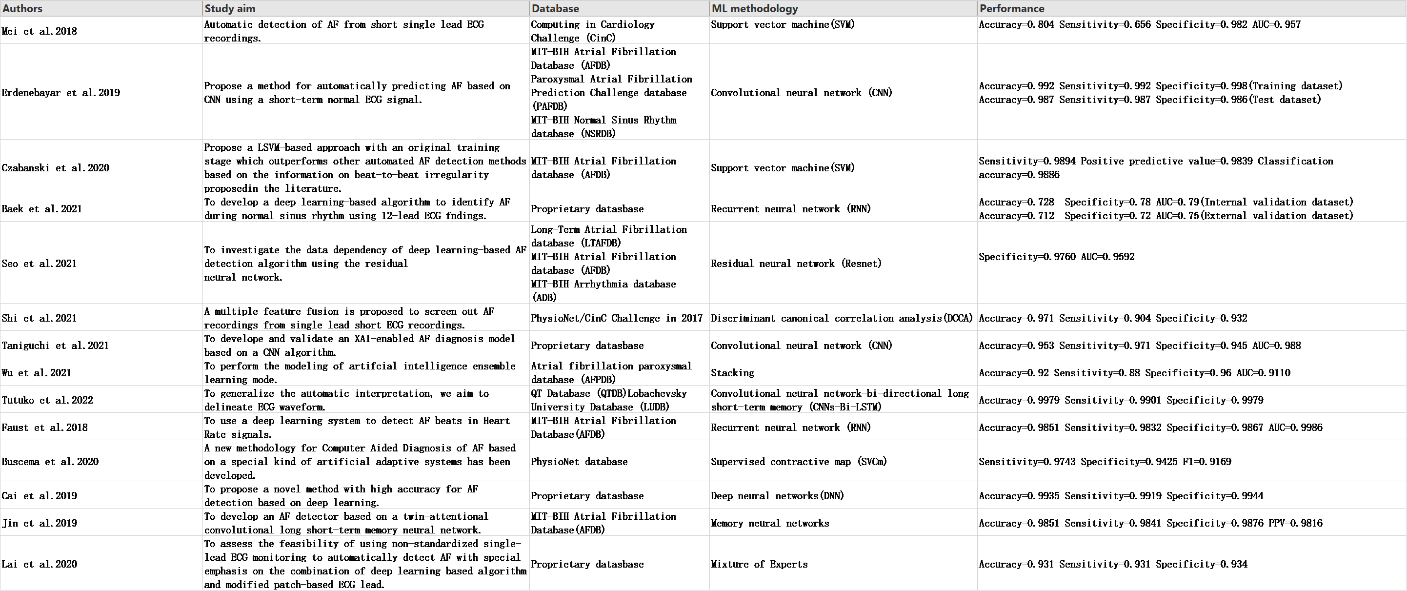


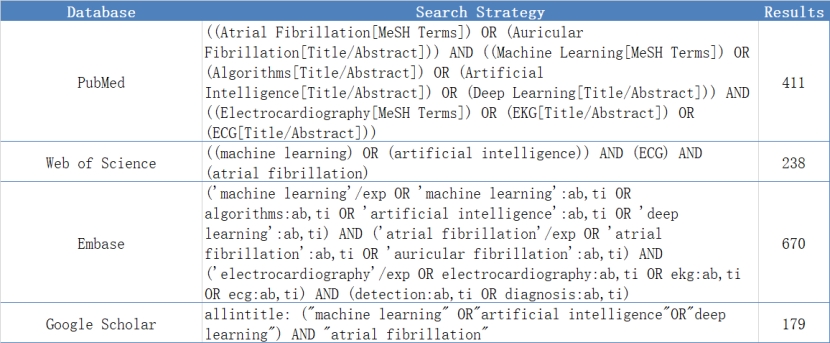


**Supplementary Fig. 1. The complete search strategy of the study.**


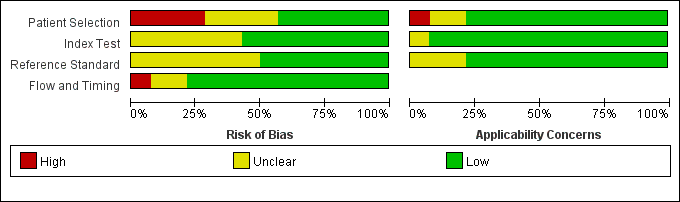


Supplementary Fig. 2. Evaluation of study quality by using the quality assessment of diagnostic accuracy studies-2 tool.
